# Efficacy of endovascular therapy for cerebral vasospasm following aneurysmal subarachnoid hemorrhage: a systematic review and meta-analysis

**DOI:** 10.3389/fneur.2024.1360511

**Published:** 2024-04-23

**Authors:** Yu-Hu Ma, Rui Shang, Si-Hao Li, Ting Wang, Sen Lin, Chang-Wei Zhang

**Affiliations:** Department of Neurosurgery, West China Hospital, Sichuan University, Chengdu, Sichuan, China

**Keywords:** aneurysmal subarachnoid hemorrhage, cerebral vasospasm, endovascular therapy, intra-arterial vasodilator infusion, meta-analysis

## Abstract

**Background:**

Cerebral vasospasm (CV) is a common complication of aneurysmal subarachnoid hemorrhage (aSAH), leading to increased morbidity and mortality rates. Endovascular therapy, particularly intra-arterial vasodilator infusion (IAVI), has emerged as a potential alternative treatment for CV.

**Methods:**

A systematic review and meta-analysis were conducted to compare the efficacy of endovascular therapy with standard treatment in patients with CV following aSAH. The primary outcomes assessed were in-hospital mortality, discharge favorable outcome, and follow-up favorable outcome. Secondary outcomes included major infarction on CT, ICU stay duration, and total hospital stay.

**Results:**

Regarding our primary outcomes of interest, patients undergoing intervention exhibited a significantly lower in-hospital mortality compared to the standard treatment group, with the intervention group having only half the mortality risk (RR = 0.49, 95% CI [0.29, 0.83], *p* = 0.008). However, there were no significant differences between the two groups in terms of discharge favorable outcome (RR = 0.99, 95% CI [0.68, 1.45], *p* = 0.963) and follow-up favorable outcome (RR = 1.09, 95% CI [0.86, 1.39], *p* = 0.485). Additionally, there was no significant difference in major infarction rates (RR = 0.79, 95% CI [0.34, 1.84], *p* = 0.588). It is important to note that patients undergoing endovascular treatment experienced longer stays in the ICU (MD = 6.07, 95% CI [1.03, 11.12], *p* = 0.018) and extended hospitalization (MD = 5.6, 95% CI [3.63, 7.56], *p* < 0.001). Subgroup analyses based on the mode of endovascular treatment further supported the benefits of IAVI in lowering in-hospital mortality (RR = 0.5, 95% CI [0.27, 0.91], *p* = 0.023).

**Conclusion:**

Endovascular therapy, particularly IAVI, holds promising potential in reducing in-hospital mortality for patients with CV following aSAH. However, it did not show significant improvement in long-term prognosis and functional recovery. Further research with larger sample sizes and randomized controlled trials is necessary to validate these findings and optimize the treatment strategy for cerebral vasospasm in aSAH patients.

**Systematic Review Registration:**

https://www.crd.york.ac.uk/PROSPERO/, identifier: CRD42023451741.

## 1 Introduction

Subarachnoid hemorrhage (SAH) is a rare subtype of stroke, constituting 2–5% of all stroke cases. Among SAH cases, 85% are attributed to the rupture of intracranial aneurysms (ICA), known as aneurysmal subarachnoid hemorrhage (aSAH) (1). The annual incidence rate of aSAH is approximately 7.9 per 100,000 individuals, with the highest occurrence observed between the ages of 40 and 60 (2–4). Despite advancements in diagnostic and treatment approaches that have led to a reduced mortality rate for aSAH, a significant proportion of survivors are left with long-term consequences (5). Cerebral vasospasm (CV) is a common complication of aSAH, typically manifesting between the 3rd and 14th day following SAH, with the highest incidence observed between the 7th and 10th day (6–8). Approximately 30–70% of aSAH patients may develop angiographic vasospasm, and among them, half may experience delayed cerebral ischemia (DCI). Ultimately, even with maximal treatment, approximately 15–20% of patients face infarction or mortality (9).

The treatment of CV remains a subject of extensive debate, with limited available treatment options (10). According to the American Heart Association (AHA) guidelines, all patients with aSAH should receive oral nimodipine (Class I; Level of Evidence A) as it reduces the risk of DCI and improves neurological outcomes. For refractory cases that do not respond to standard treatment, AHA suggests that endovascular therapy may be a reasonable option (Class IIa; Level of Evidence B) (11). Over time, endovascular therapy has gained widespread clinical application and has emerged as an alternative treatment for CV, challenging the traditional standard therapy (12–14). Endovascular therapy encompasses two methods: transluminal balloon angioplasty (TBA) and intra-arterial vasodilator infusion (IAV/IAVI). TBA is suitable for acute-phase CV and provides rapid and direct effects, but is limited to proximal vessels and focal cerebral vasospasm (15, 16). IAVI is another commonly used endovascular treatment for CV, involving the direct delivery of vasodilatory drugs into the spastic vessel via a catheter. This approach aims to dilate the narrowed vessel and increase cerebral blood flow. Commonly used drugs include nitroprusside, calcium channel blockers (such as nimodipine, nicardipine, and verapamil), and the selective phosphodiesterase-3 inhibitor milrinone, et al. (17–19). IAVI can be employed during the acute or subacute phase and is suitable for managing more distal and diffuse vessel spasms.

Currently, the superiority of endovascular therapy over standard treatment remains a subject of controversy, despite it being considered as a salvage treatment after standard therapy failure. The clinical literature reports considerable variation in the outcomes. Given these discrepancies, we conducted a systematic review and meta-analysis to compare the effects of endovascular therapy and standard treatment on post-aSAH cerebral vasospasm. The objective is to provide recommendations for clinicians in selecting treatment strategies and offer guidance for the design of future clinical trial protocols.

## 2 Materials and methods

### 2.1 Protocol and registration

This meta-analysis adhered to the recommendations and guidelines outlined in the Preferred Reporting Items for Systematic Reviews and Meta-Analyses (PRISMA) (20). The protocol for this study was registered on PROSPERO. Ethical approval was not necessary for this work as it involved the analysis of previously published data.

### 2.2 Eligibility criteria

The PICOS statements of this study are as follows:

#### 2.2.1 P(Population)

The study population comprised adult patients (aged over 18 years) with cerebral vasospasm following aSAH. CV diagnosis was based on hemodynamic and imaging criteria. Initially indicated by transcranial Doppler sonography (TCD) showing a mean flow velocity over 160 cm/s in the anterior circulation or an increase exceeding 50% within 24 h, CV was suspected particularly when associated with unexplained neurological symptoms. For definitive diagnosis, imaging techniques such as computed tomography angiography (CTA) and digital subtraction angiography (DSA) were employed, confirming CV through findings of more than 50% vessel diameter reduction or the presence of vascular irregularities.

#### 2.2.2 I(Intervention)

Endovascular treatment, specifically IAVI and TBA.

#### 2.2.3 C(Comparison)

Standard treatment, including oral or intravenous vasodilator infusion and Triple-H therapy, etc.

#### 2.2.4 O(Outcome)

Include one of the following primary outcomes at least:

Primary outcomes:

1. Favorable outcomes in discharge and follow-up for more than 3 months. The prognosis of patients was evaluated using the Glasgow Outcome Scale (GOS) and the Modified Rankin Scale (mRS). Favorable outcomes were defined as a GOS score of ≥4 or an mRS score of ≤2.
2. In-hospital mortality.

Additionally, the study examined the following secondary outcomes:

1. Cerebral infarction: Infarction detected on CT at discharge.
2. Relevant time information during hospitalization, such as intensive care unit (ICU) stay times and total hospital days.

#### 2.2.5 S(Study design)

This study included randomized controlled trials (RCTs) and retrospective cohort studies. If propensity-matched analyses (PSM) were conducted, preference was given to studies with PSM data.

We specifically included the studies comparing the efficacy of endovascular treatment and standard treatment in patients with CV. Studies that solely reported the prognosis of patients treated with endovascular therapy for CV were excluded.

### 2.3 Search and study selection

A systematic literature search was performed on PubMed, The Cochrane Library, Web of Science, and EMBASE to identify relevant studies on cerebral vasospasm up to January 1, 2023. Additionally, a snowball method was utilized to search for references cited in the included literature to identify additional studies that met the eligibility criteria. The detailed search strategies used in PubMed can be found in Supplementary Table S1.

### 2.4 Study selection

Two review authors independently screened the titles and abstracts of the identified literature using the predetermined PICOS criteria to exclude clearly unrelated reports. Subsequently, they thoroughly read the full text of the remaining articles to determine which studies met the inclusion criteria. Any studies that did not meet the inclusion requirements were recorded along with the reasons for their exclusion. In cases where there were disagreements in judgment, the two reviewers engaged in discussions to reach a consensus. If any disagreements persisted, a third review author was involved in the discussion to facilitate resolution. The selection process was documented, and a PRISMA flow diagram was completed to illustrate the study selection procedure.

### 2.5 Data collection

Two review authors independently extracted the following data from the included studies and recorded the information on the data extraction table using Microsoft Office Excel 2019. The following data were extracted:

(1) Basic information on included studies: first author, year of publication, study design and follow-up time, etc. (2) Baseline characteristics of the study queue, such as type of intervention group, sample size, Hunt-Hess score, etc. (3) The primary and secondary outcomes of interest in this study.

Any disagreements were resolved by discussion between the two review authors.

### 2.6 Assessment of risk of bias in included studies

The risk of bias in each study was assessed using the Newcastle-Ottawa Scale (NOS), a validated tool. The scale assigns a score ranging from 0 to 9, with a score of ≥7 indicating a study with a low risk of bias (21, 22). Two review authors independently evaluated the risk of bias for each study. In instances where discrepancies occurred, they engaged in discussions to reach a consensus. If an agreement could not be reached, a third reviewer participated in the deliberations until a resolution was achieved.

### 2.7 Statistical analysis

All data analyses were performed using Review Manager (version 5.3, Cochrane Collaboration) and Stata (version 16, StataCorp, College Station, Texas) software. Binary variables were expressed as risk ratios (RR), continuous variables with consistent measurement units were presented as mean differences (MD), and standardized mean differences (SMD) were used for variables with inconsistent measurement units. When necessary, estimation of means and standard deviations was conducted using an online tool based on median, range, and sample size, as described by Wan et al. (23). Subgroup analyses were planned based on specific intervention measures. All variables were calculated with 95% confidence intervals (CI). All reported *p*-values were two-sided, and *p*-values <0.05 were considered statistically significant. Heterogeneity of the included studies was assessed using the Q test and I^2^ test. Due to the limited number of studies included, we have decided to adopt a random effects model for the analysis of all results. We conducted sensitivity analysis on results with significant heterogeneity (i.e., I^2^ ≥ 50%) to investigate the impact of individual studies on overall effectiveness estimation. If at least five studies were available for the outcomes of interest, visual funnel plot analysis was conducted to assess publication bias, with greater asymmetry indicating a higher likelihood of substantial bias. Additionally, Egger’s and Harbord’s tests were used to assess publication bias.

## 3 Results

### 3.1 Characteristics of the included studies

**Figure 1:**
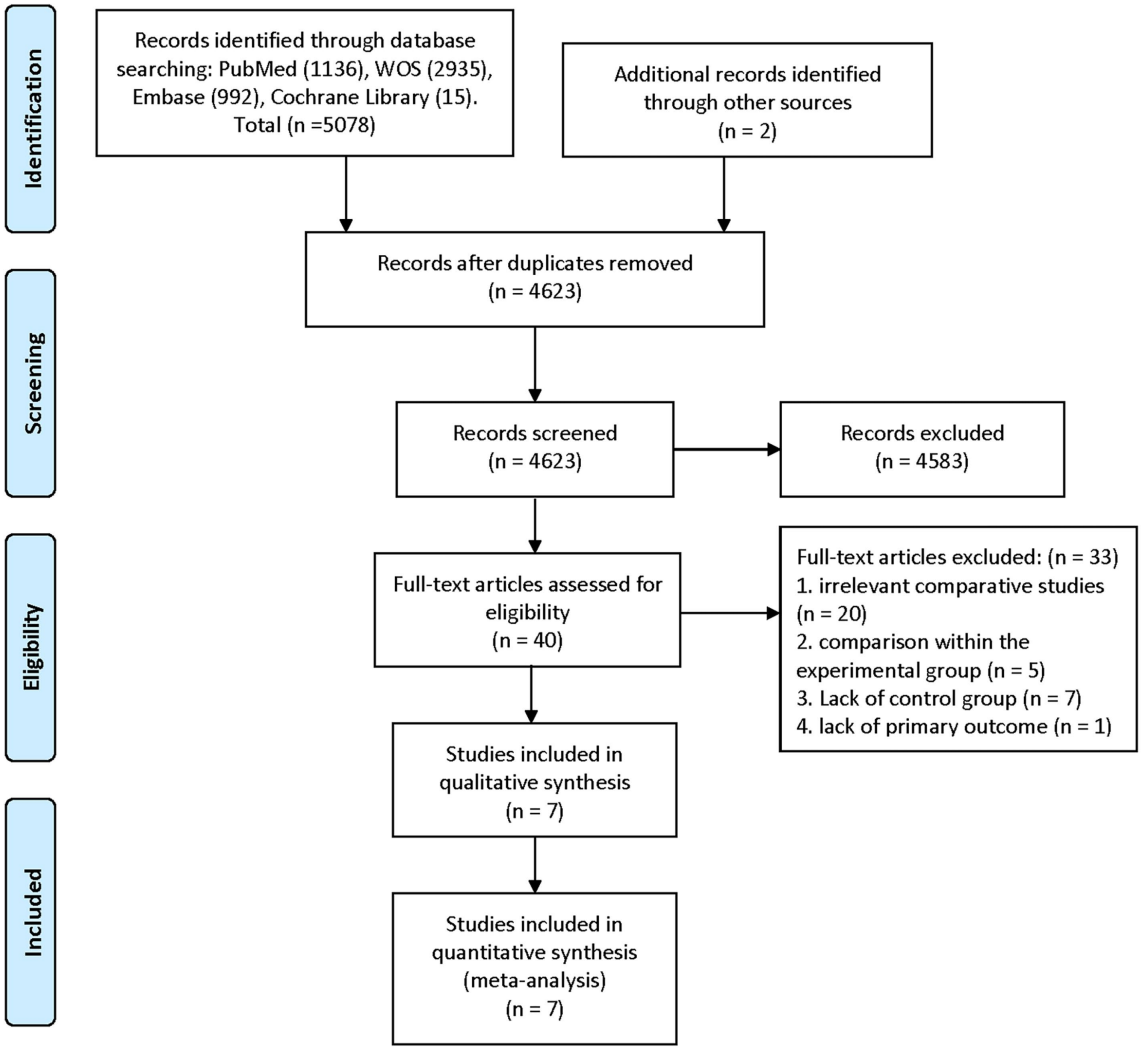
Flowchart of study selection for the present study.

**Figure 2:**
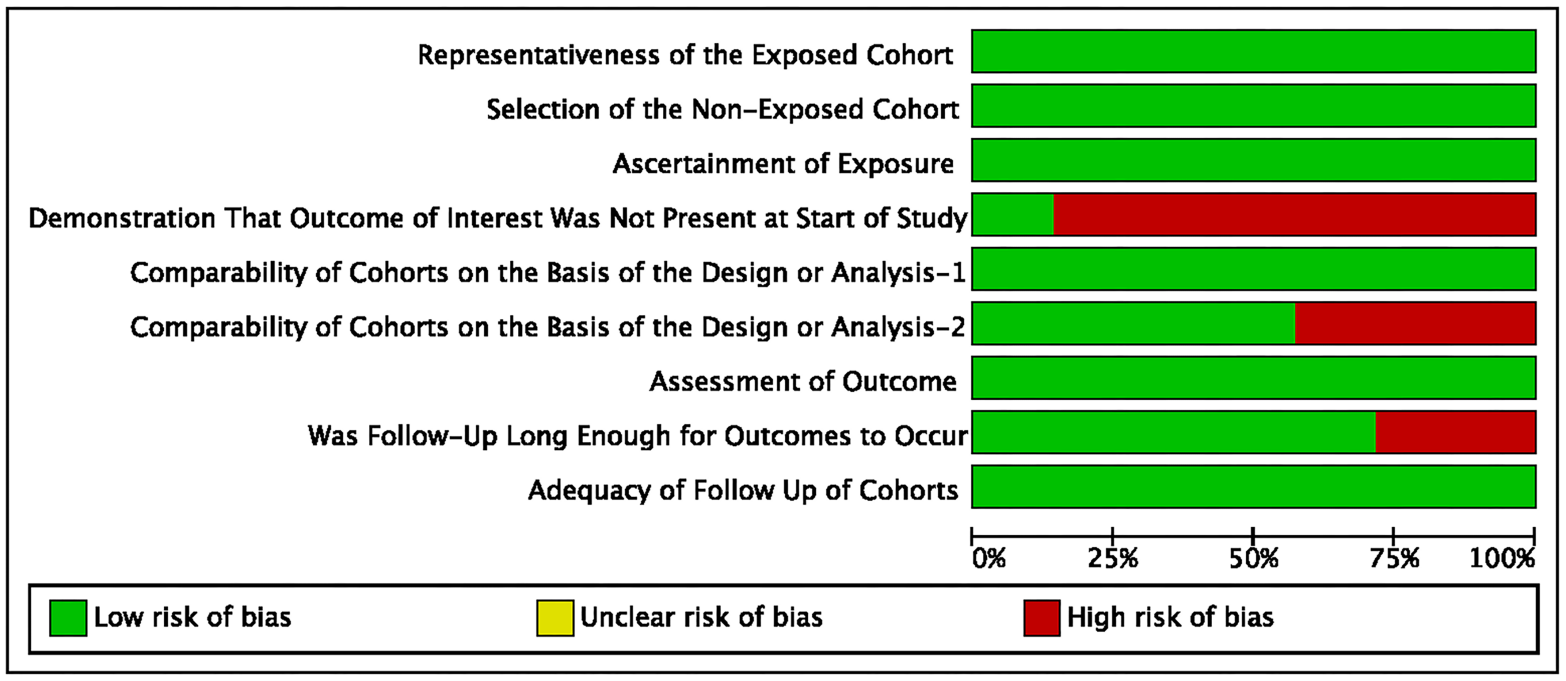
Risk of bias graph: results of NOS in included studies.

**Figure 3:**
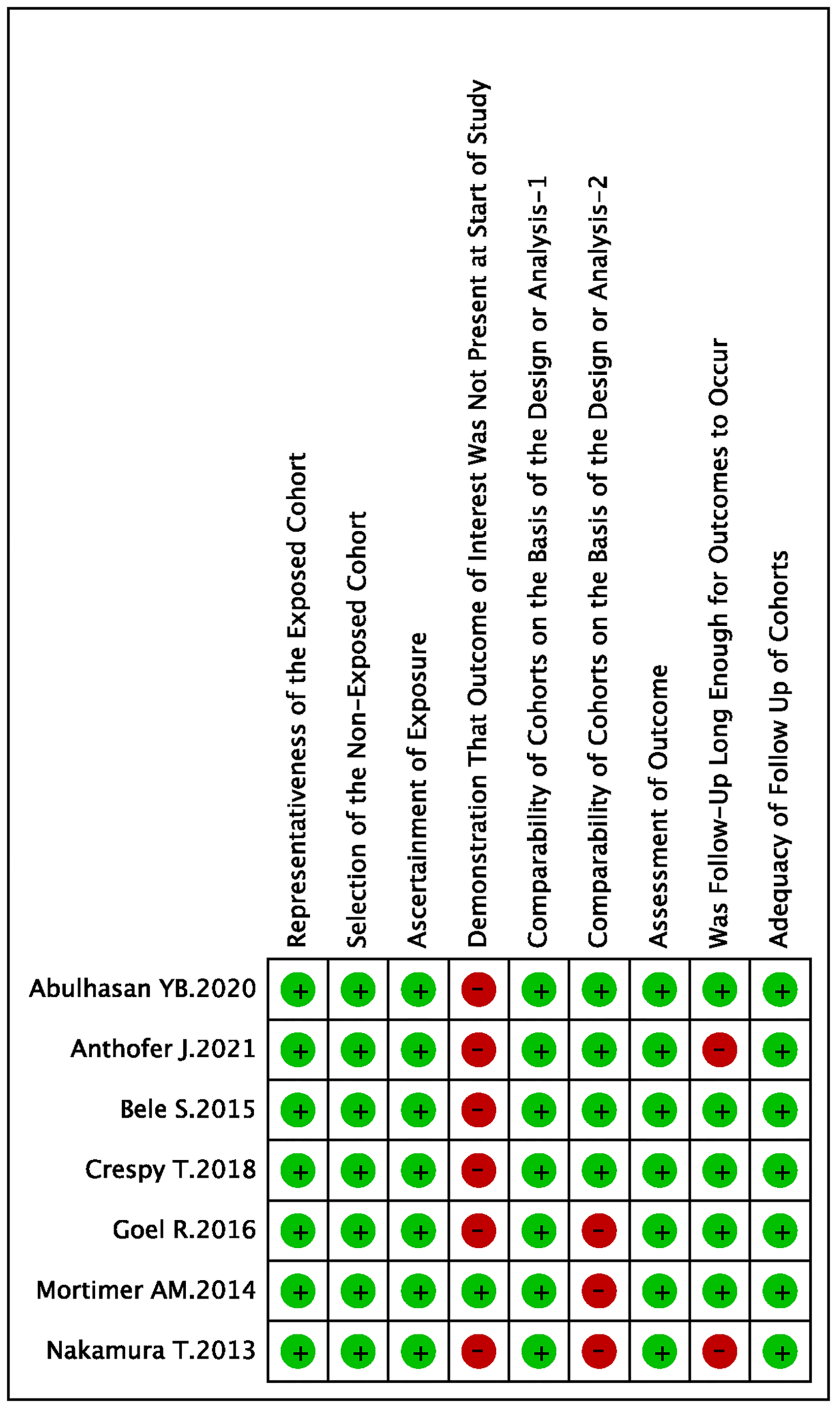
Risk of bias summary: judgements about each risk of bias item for each included study.

The summary of the search results is presented in a PRISMA study flow diagram (Figure 1). A comprehensive literature search yielded a total of 5,078 articles, which were subsequently refined to 4,623 after eliminating duplicate entries. Through meticulous scrutiny of titles and abstracts, we excluded 4,583 papers that were deemed irrelevant, enabling us to proceed with a detailed examination of the complete texts of the remaining 40 studies. Nonetheless, 33 articles were excluded from our analysis for the following reasons: 20 studies involved irrelevant comparative analyses, five studies focused on comparisons within the experimental group, seven studies lacked a control group, and one study did not report the primary outcome. Ultimately, our systematic review and meta-analysis included seven studies (24–30) that satisfied the predetermined inclusion criteria. No randomized controlled trials (RCTs) were identified among the included studies. Among the selected studies, one (29) employed a prospective cohort design, while the remaining six studies were retrospective cohort studies. Five studies (24, 26–28, 30) employed endovascular treatment using IAV, while two studies (25, 29) employed a combination of IAV and TBA treatment. Two studies (24, 30) provided data for two distinct groups. Table 1 provides a comprehensive summary of the characteristics of all the articles included in our analysis. Each of the included studies underwent an assessment of bias risk using the NOS tool. The scores assigned ranged from 8, 7, to 6, with four studies, two studies, and one study achieving these respective scores. The average score across the studies was 7.4. The results of the bias risk assessment are graphically depicted in Figures 2, 3 (High risk: 0 score, Low risk: 1 score).

**Table 1:** Baseline characteristics of individual studies.

Author	Year	Country	Design	Total (*n*)	Intervention (*n*)	Control (*n*)	Type of intervention group	Type of control group	Outcome evaluation	Quality score
Anthofer J-1	2021	Germany	RS	52	15	37	Single shot IA nimodipine	HHT/NHT and oral nimodipine	GOS	7
Anthofer J-2	2021	Germany	RS	86	49	37	Continuous IA nimodipine	HHT/NHT and oral nimodipine	GOS	
Abulhasan YB	2020	Canada	RS	110	21	89	TBA and/or IA milrinone	IV milrinone	mRS	8
Crespy T	2018	France	RS	101	24	77	Continuous IA milrinone	Continuous IV milrinone	/	8
Goel R	2016	India	RS	53	39	14	IA nimodipine	Oral nimodipine	GOS	7
Bele S	2015	Germany	RS	41	21	20	continuous IA nimodipine	HHT and oral nimodipine	GOS	8
Mortimer AM	2014	Australia	PS	80	17	63	TBA and/or IA verapamil and papaverine	IV nimodipine	GOS and mRS	8
Nakamura T-1	2013	Japan	RS	21	10	11	Selective IA FA	IV FA	GOS	6
Nakamura T-2	2013	Japan	RS	21	10	11	Nonselective IA FA	IV FA	GOS	

RS: retrospective, PS: prospective, IA: intraarterial, IV: intravenous, TBA: transluminal balloon angioplasty, FA: fasudil hydrochloride, HHT: hypervolemic hypertension therapy, NHT: normovolemic hypertension therapy, GOS: glasgow outcome scale, mRS: modified rankin scale.

### 3.2 Baseline characteristic analysis

A total of 517 participants were included in the meta-analysis, with 206 individuals allocated to the intervention group and 311 individuals to the control group. Comprehensive baseline information is presented in Supplementary Figures S1, S2. The assessment of neurological status upon admission for all patients primarily relied on the World Federation of Neurosurgical Societies (WFNS) Score, Modified Fisher Score, and Hunt-Hess Score. The study findings revealed no significant disparities in the neurological status at admission between the two groups. However, it was observed that the intervention group had a statistically significant mean age difference of 3.64 years younger compared to the control group (MD = -3.64, 95% CI [−6.01, −1.27], *p* < 0.05). Notwithstanding this age difference, all other baseline characteristics between the two groups demonstrated comparability.

### 3.3 Perioperative characteristics and outcomes

**Figure 4:**
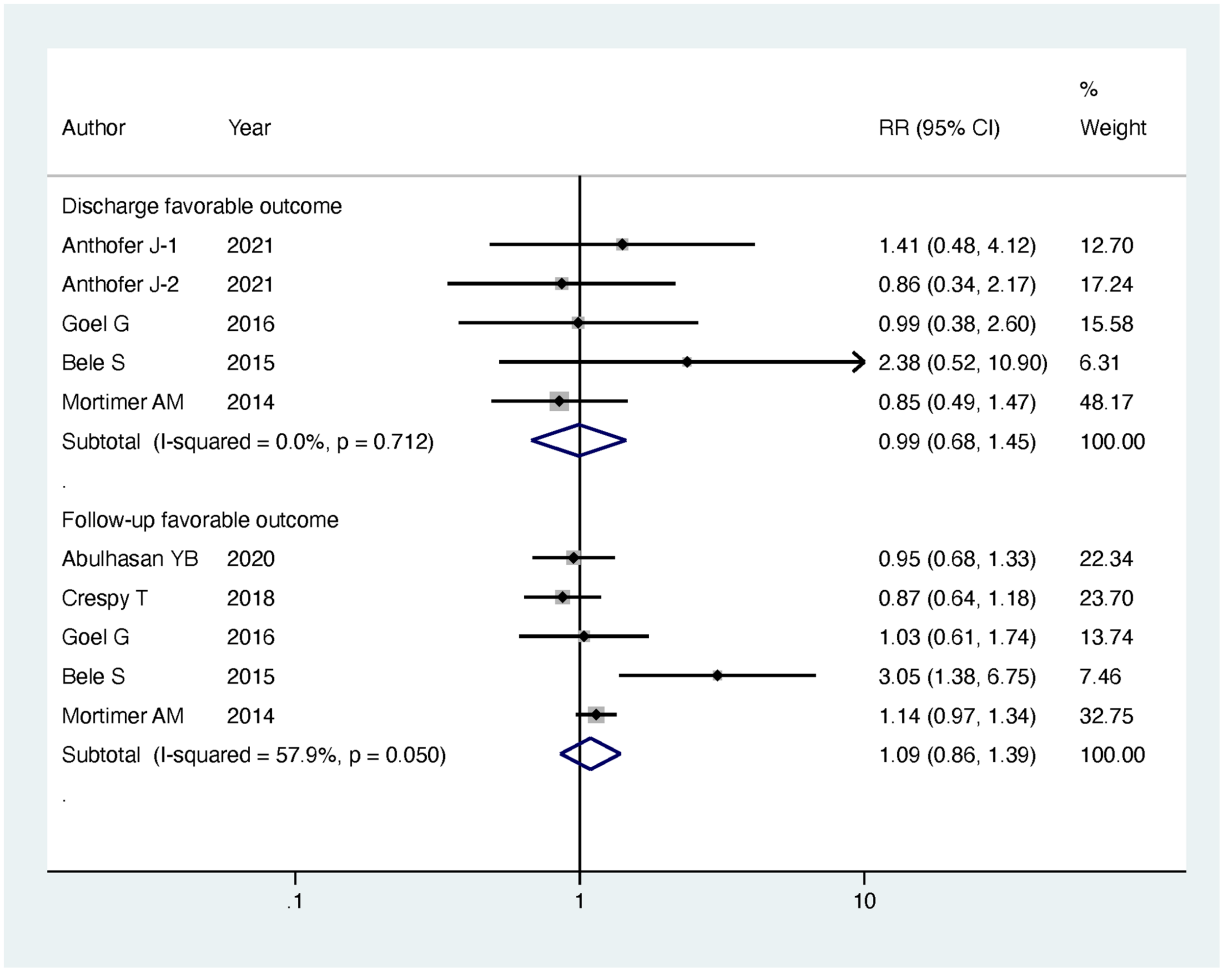
Meta-analysis of favorable outcomes in discharge and follow-up for more than 3 months.

**Figure 5:**
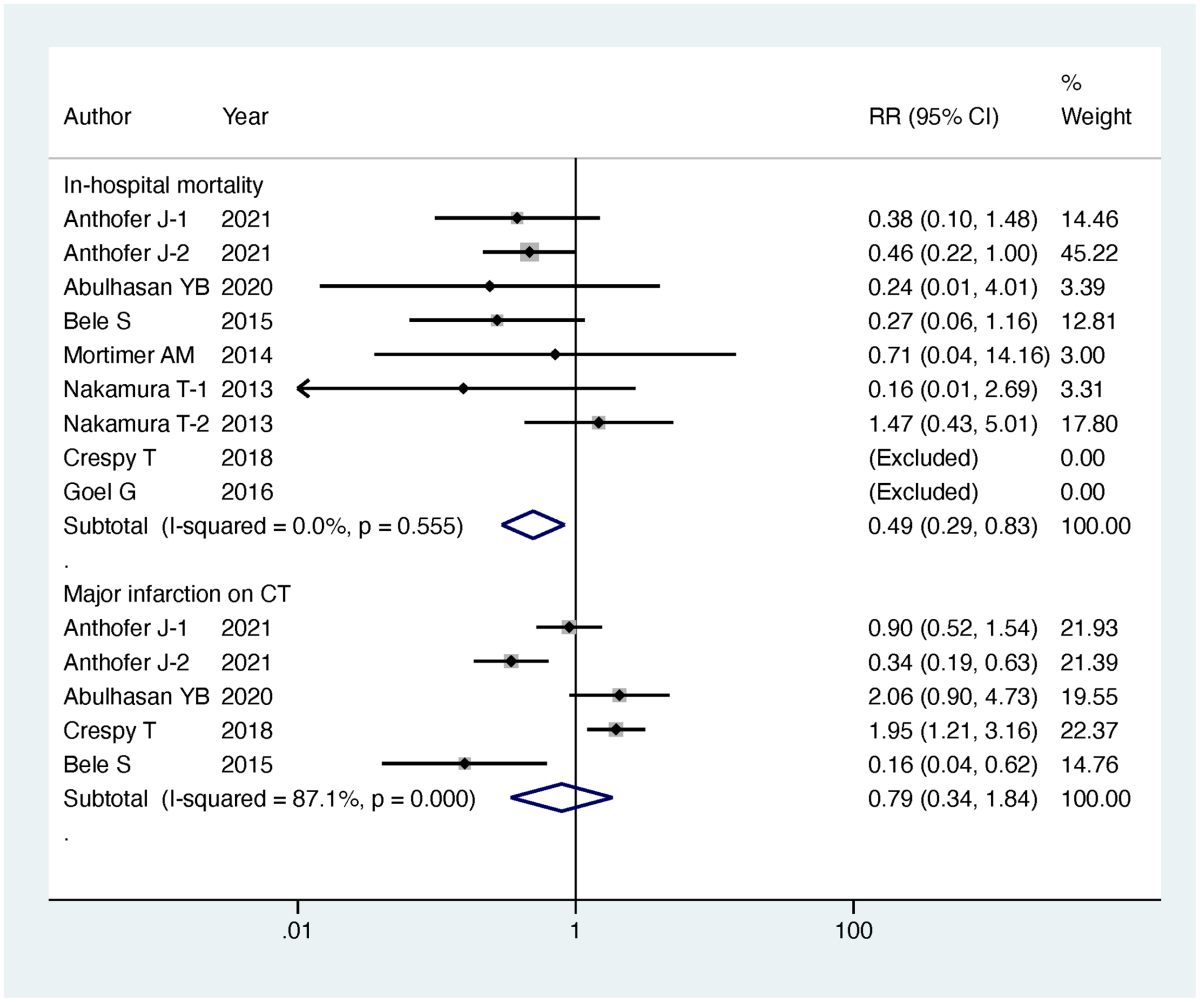
Meta-analysis of in-hospital mortality and cerebral infarction.

**Figure 6:**
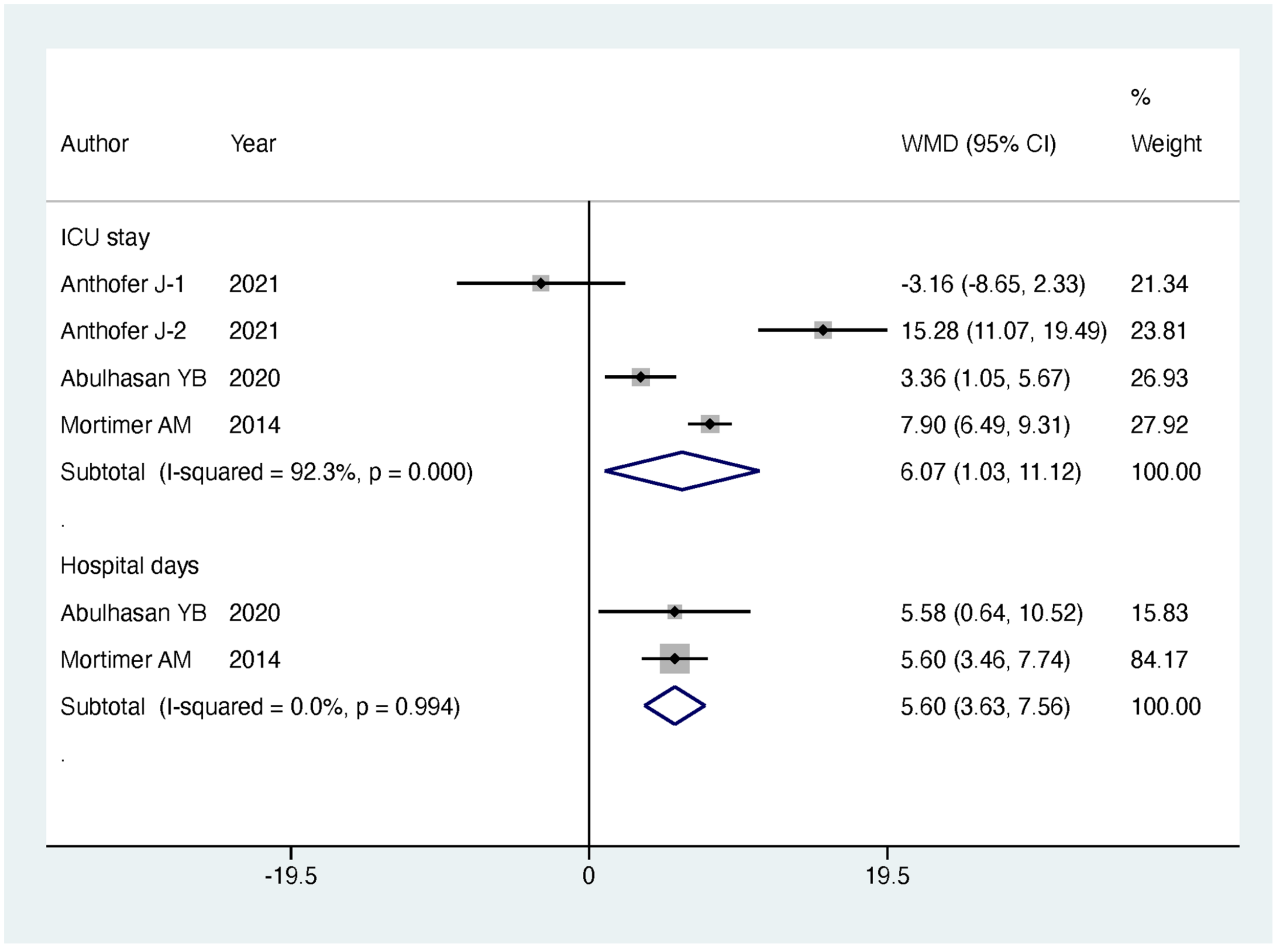
Meta-analysis of ICU stay times and total hospital days.

Regarding our primary outcomes of interest, patients undergoing endovascular intervention exhibited a significantly lower in-hospital mortality compared to the standard treatment group, with the intervention group having only half the mortality risk (RR = 0.49, 95% CI [0.29, 0.83], *p* = 0.008). However, there were no significant differences between the two groups in terms of discharge favorable outcome (RR = 0.99, 95% CI [0.68, 1.45], *p* = 0.963) and follow-up favorable outcome (RR = 1.09, 95% CI [0.86, 1.39], *p* = 0.485). Regarding secondary outcomes, there was no significant difference observed in major infarction between patients receiving endovascular intervention and those receiving standard treatment (RR = 0.79, 95% CI [0.34, 1.84], *p* = 0.588). However, patients undergoing endovascular treatment experienced significantly longer stays in the intensive care unit (ICU) (MD = 6.07, 95% CI [1.03, 11.12], *p* = 0.018) and more extended hospitalization (MD = 5.6, 95% CI [3.63, 7.56], *p* < 0.001). The detailed results can be found in Table 2, and the forest plots are provided in Figures 4–6.

**Table 2:** Pooled results of primary and secondary outcomes of the meta-analysis.

Outcomes	No. of studies	Total No. of patients	Effect measure	Pooled results				Heterogeneity		*p* value for Egger’s test	*p* value for Harbord’s test
				Effect estimate	LCI	UCI	*p* value	I^2^ (%)	*p* value		
Primary outcomes											
Discharge favorable outcome	5	275	RR	0.99	0.68	1.45	0.963	0	0.712	0.008	0.011
Follow-up favorable outcome	5	383	RR	1.09	0.86	1.39	0.485	57.9	0.05	0.191	0.318
In-hospital mortality	7	517	RR	0.49	0.29	0.83	0.008	0	0.555	0.896	0.81
Secondary outcomes											
Major infarction on CT	5	347	RR	0.79	0.34	1.84	0.588	87.1	<0.001	0.506	0.916
ICU stay/days	4	291	MD	6.07	1.03	11.12	0.018	92.3	<0.001	/	/
Hospital days	2	190	MD	5.6	3.63	7.56	<0.001	0	0.994	/	/

CT: computed tomography, ICU: intensive care unit, RR: risk ratios, MD: mean differences, LCI: lower confidence interval, UCI: upper confidence interval.

To gain further insights, we conducted subgroup analyses based on the mode of endovascular treatment. The results showed that patients treated with IAV had a significantly lower in-hospital mortality compared to the standard treatment group, with the risk being half (RR = 0.5, 95% CI [0.27, 0.91], *p* = 0.023). In contrast, there was no significant difference in in-hospital mortality between patients receiving both IAVI and TBA and the standard treatment group (RR = 0.4, 95% CI [0.05, 3.11], *p* = 0.381). Moreover, subgroup analysis revealed that the differences in ICU stay and hospital days between the intervention and control groups were mainly driven by patients receiving both IAVI and TBA, while patients undergoing IAV treatment did not show significant differences in ICU stay and hospital days compared to those receiving standard treatment. Furthermore, the results of subgroup analysis indicated no significant differences in discharge favorable outcome, follow-up favorable outcome, and major infarction between patients receiving either IAV or receiving both IAVI and TBA, yielding nonsignificant results. Detailed results of the subgroup analysis are presented in Table 3, and the funnel plots for the subgroup analysis can be found in Supplementary Figures S3–S7.

**Table 3:** Pooled results of subgroup analyses of the outcomes.

Subgroup outcomes		No. of studies	Effect measure	Pooled results				Heterogeneity	
				Effect estimate	LCI	UCI	*p* value	I^2^ (%)	*p* value
Primary outcomes									
Discharge favorable outcome	IAV	4	RR	1.15	0.67	1.95	0.613	0	0.684
	IAV combination TBA	1	RR	0.85	0.49	1.47	0.555	0	0.712
Follow-up favorable outcome	IAV	3	RR	1.28	0.67	2.43	0.45	78.3	0.01
	IAV combination TBA	2	RR	1.08	0.88	1.32	0.446	30.7	0.23
In-hospital mortality	IAV	7	RR	0.5	0.27	0.91	0.023	12.4	0.335
	IAV combination TBA	2	RR	0.4	0.05	3.11	0.381	0	0.597
Secondary outcomes									
Major infarction on CT	IAV	4	RR	0.62	0.23	1.67	0.346	89.2	<0.001
	IAV combination TBA	1	RR	2.06	0.9	4.73	0.087	/	/
ICU stay/days	IAV	2	MD	6.15	−11.92	24.22	0.505	96.3	<0.001
	IAV combination TBA	2	MD	5.73	1.28	10.17	0.012	90.7	0.001
Hospital days	IAV	0	MD	/	/	/	/	/	/
	IAV combination TBA	2	MD	5.6	3.63	7.56	<0.001	0	0.994

IAV: intra-arterial vasodilator infusion, TBA: transluminal balloon angioplasty, CT: computed tomography, ICU: intensive care unit, RR: risk ratios, MD: mean differences, LCI: lower confidence interval, UCI: upper confidence interval.

### 3.4 Sensitivity analysis and publication bias

During the comprehensive analysis of the results, we observed substantial heterogeneity in three outcomes, namely Follow-up favorable outcome, Major infarction on CT, and ICU stay, with an I^2^ value exceeding 50%. To further investigate these outcomes, we conducted sensitivity analyses. Remarkably, the sensitivity analysis results revealed that the statistical significance of the combined estimates remained consistent across all outcomes.

Moreover, we performed an assessment of publication bias for four outcomes that included five or more studies: Discharge favorable outcome, Follow-up favorable outcome, In-hospital mortality, and Major infarction on CT. Egger’s test and Harbord’s test highlighted the presence of publication bias in relation to Discharge favorable outcome, while the risk of publication bias for the remaining three outcomes was determined to be low. Detailed results can be found in Table 2. For a visual representation of the publication bias assessment, please refer to Supplementary Figures S8–S11, displaying the funnel plots.

## 4 Discussion

Cerebral vasospasm is a prevalent and severe complication following aneurysmal subarachnoid hemorrhage (1, 31, 32). This condition imposes a significant burden on patients’ prognosis and economic status, with studies reporting that 25–50% of patients may experience disabilities, while only 30–45% are able to recover to their previous work status (33). Additionally, patients undergoing treatment for CV face an additional economic burden of up to 30% compared to those without CV (34). Hence, timely and effective treatment for cerebral vasospasm is crucial in improving patients’ prognosis and quality of life. Currently, there is no consensus on the treatment approach for CV in the existing treatment guidelines. Therefore, we conducted this meta-analysis to assess the efficacy and safety of endovascular therapy compared to standard treatment in patients with CV. Our objective is to furnish novel insights and recommendations for the management of CV through this meta-analysis, ultimately aiming to enhance patients’ recovery and survival outcomes in the future.

The results of this study indicate that endovascular therapy leads to a perioperative mortality rate that is only half of that observed with standard treatment. Subgroup analysis reveals that this outcome is primarily driven by the IAVI group, where patients receiving both IAVI and TBA exhibit no significant difference in in-hospital mortality compared to standard treatment. This highlights the significant advantage of IAVI in reducing the risk of in-hospital mortality, effectively halving the rate and holding crucial implications for saving patients’ lives. However, it is noteworthy that no significant differences are observed between the IAVI and standard treatment groups concerning the discharge favorable outcomes and follow-up favorable outcomes. In secondary outcomes, patients receiving IAVI show no significant differences in terms of major infarction, ICU stay duration, and total hospital stay when compared to those undergoing standard treatments. This indicates that, although IAVI can reduce perioperative mortality compared to standard treatment, it does not significantly improve patients’ recovery and long-term prognosis, nor does it completely eliminate or reduce the risk of severe infarctions. Nevertheless, this does not imply that IAVI lacks potential benefits. Other factors may have contributed to these results, such as insufficient sample size or inadequate follow-up duration. Furthermore, patients’ recovery and prognosis may be influenced by multiple factors beyond the treatment method. Despite the lack of long-term advantages in prognosis, the clear short-term efficacy of IAVI is evident. Its ability to lower in-hospital mortality is undeniably beneficial for patients and represents a critical clinical achievement, leading us to recommend IAVI as a valuable option for treating cerebral vasospasm. On the other hand, the neurological functional recovery provided by IAVI for cerebral vasospasm is limited, necessitating its combination with other rehabilitation and support measures to optimize treatment strategies and improve patients’ quality of life and functional recovery.

Currently, there is significant controversy in the clinical setting regarding the effectiveness of TBA treatment for CV (20, 35). Some studies have conducted retrospective evaluations to explore the feasibility and efficacy of TBA treatment. Tsogkas et al. (36) conducted a study retrospectively evaluating 17 patients who received Scepter C balloon catheter treatment between 2014 and 2018. These patients were successfully treated for CV after failed medical therapy, with no associated complications. Some researchers have also conducted a similar study, demonstrating the feasibility of Scepter C balloon catheter treatment for CV (37). Another research supports TBA as a therapeutic option for severe proximal vessel spasm and reduced tissue perfusion and suggests considering more frequent use of TBA in conjunction with PW/DW imaging, rather than solely as a last-resort treatment measure (38). However, existing research only provides single-group follow-up results for TBA treatment in CV, and there have been no cohort studies in the clinical setting to compare the differences between TBA and standard treatment. The subgroup analysis in this meta-analysis reveals that receiving both IAVI and TBA only prolongs patients’ ICU stay duration and total hospital stay, with no apparent benefit in terms of therapeutic efficacy for CV compared to the standard treatment group. Our research findings did not indicate a beneficial effect of TBA on patients’ prognosis, but it is important to note that this conclusion is based on the results of only two studies. In the future, high-quality, large-scale clinical studies are still needed to provide compelling evidence regarding the effectiveness of TBA in treating CV. It is noteworthy that the combination therapy of IAVI with TBA demonstrated lower efficacy in improving primary outcomes compared to IAVI monotherapy. This discrepancy may be attributed to multiple factors. Firstly, the increased procedural complexity and the risk of complications associated with dual therapy might negate the individual advantages of IAVI and TBA, especially in patients with complex vascular anatomy or severe vasospasm. Secondly, the timing and criteria for selecting patients for combined therapy versus monotherapy could influence treatment outcomes, as those undergoing combined treatment may have more severe or intractable vasospasm, leading to inferior results. Additionally, the variation in technical expertise and procedural practices across different treatment centers could impact the success rates and clinical outcomes of these therapies. Therefore, conducting further research is essential to gain a more accurate understanding of the optimal timing and patient selection for each treatment modality.

Our meta-analysis revealed nuanced outcomes, with no clear differences between endovascular therapy and standard care, highlighting the need for an in-depth analysis. Contributing to this are the diverse patient demographics and the variability in both the severity and timing of CV treatment, which may obscure outcome disparities. The range of endovascular techniques applied, encompassing various devices and procedural nuances, could lead to outcomes that do not decisively favor one treatment approach over another. Additionally, inconsistencies in clinical practices among different treatment centers and the absence of standardized protocols for CV management could lead to uneven results. The timing of the intervention relative to symptom onset also plays a pivotal role; if not optimally aligned within the therapeutic window, it may result in subdued treatment benefits. These aspects highlight the need for meticulous design in future studies to clarify the effects of endovascular therapies on CV, emphasizing the importance of standardized treatment approaches and optimal timing of intervention.

There are several methods used for the prevention of CV caused by aSAH. The widely used “triple-H” therapy, which includes induced hypertension, hypervolemia, and hemodilution, was once commonly employed. However, research failed to demonstrate the benefits of prophylactic triple-H treatment, possibly due to its association with other complications, leading to its current disuse. Presently, oral nimodipine is the only treatment method recommended in guidelines for all aSAH patients. Although it does not improve CV, it has been proven effective in enhancing neurological outcomes. Other prophylactic uses of CCB, corticosteroids, and magnesium sulfate have not shown clinical significance (11, 39). Current guidelines do not recommend prophylactic hypervolemia or TBA before angiographic spasm occurs. Research results on TBA are inconsistent, with some studies indicating a reduced risk of DCI and CV recurrence, but failing to demonstrate clinical benefits (40, 41). Some studies suggest that intraventricular drainage may have application value in preventing CV, especially in reducing the incidence of vasospasm, mortality rate, and the need for endovascular rescue therapy following procedures such as thrombus removal and ventriculostomy (42–44). Overall, prophylactic treatment should still be chosen based on physicians’ experience and treatment center conditions. Postoperative measures such as active blood volume management, monitoring of patients’ neurological status, enhanced imaging review, and continued oral nimodipine administration remain relatively recommended preventive measures. Recent evidences indicate that several diagnostic tools, such as perfusion CT scan, continuous electroencephalography, xenon-CT scan and brain microdialysis and elevated CSF lactate and glucose levels in the first 3 days following aSAH are predictors of subsequent DCI-related neurological impairment (45–48). Further research is still necessary to determine the most effective preventive approaches.

## 5 Limitation

This study encompasses certain limitations that may affect the interpretation of results. Primarily, it includes only 7 studies, each with limited sample sizes, which may not adequately represent the broader patient population, thereby necessitating larger-scale studies for more definitive validation. These studies originate from diverse clinical centers across different countries, introducing potential variability in data source processing and treatment protocols. Moreover, the generally short follow-up period in these studies restricts our ability to comprehensively analyze long-term outcomes post-discharge. Although we endeavored to apply unified evaluation criteria to mitigate the impact of heterogeneity, the variability in treatment approaches, timing, and endovascular techniques used across different centers remains a challenge. Notably, the lack of RCTs addressing this specific condition underscores a significant gap in high-quality evidence. Therefore, future research needs to overcome these limitations to improve the reliability and generalizability of the study.

## 6 Conclusion

Endovascular therapy, especially IAVI, is a promising treatment option for reducing in-hospital mortality in patients with CV. However, no significant improvement has been observed in long-term prognosis and functional recovery. Further research with larger sample sizes and randomized controlled trials is necessary to validate the study findings and optimize the treatment strategy for cerebral vasospasm in aSAH patients.

## Data availability statement

The original contributions presented in the study are included in the article/Supplementary material, further inquiries can be directed to the corresponding author.

## Author contributions

Y-HM: Conceptualization, Data curation, Formal analysis, Funding acquisition, Investigation, Methodology, Project administration, Resources, Software, Supervision, Validation, Visualization, Writing – original draft, Writing – review & editing. RS: Data curation, Investigation, Methodology, Software, Supervision, Writing – original draft. S-HL: Data curation, Formal analysis, Methodology, Software, Writing – review & editing. TW: Data curation, Formal analysis, Investigation, Methodology, Software, Writing – review & editing. SL: Formal analysis, Investigation, Software, Writing – review & editing. C-WZ: Conceptualization, Data curation, Formal analysis, Funding acquisition, Investigation, Methodology, Project administration, Resources, Software, Supervision, Validation, Visualization, Writing – original draft, Writing – review & editing.
