# Supplementary material for: Efficacy of endovascular therapy for cerebral vasospasm following aneurysmal subarachnoid hemorrhage: a systematic review and meta-analysis
Source: Front Neurol. 2024 Apr 23;15:1360511. doi: 10.3389/fneur.2024.1360511 (PMC11075757; doi:10.3389/fneur.2024.1360511)
Supplement: Supplementary file 1 [file Data_Sheet_1.doc]

| **Set** | **Quary** |
| --- | --- |
| **#1** | "Subarachnoid Hemorrhage"[Mesh] |
| **#2**  **#3** | "subarachnoid hemorrhage"  SAH OR subarachnoid OR blood OR hemorrhage |
| **#4** | #1 OR #2 OR #3 |
| **#5** | "Vasospasm, Intracranial"[Mesh] |
| **#6** | "Intracranial Vasospasm" OR "Cerebral Vasospasm" OR vasospasm |
| **#7** | "Intracranial Angiospasm" OR "Cerebral Angiospasm" OR angiospasm |
| **#8**  **#9** | "Intracranial Vascular Spasm" OR "Cerebral Artery Spasm"  "Cerebrovascular Spasm" OR "brain vasospasm" OR vasoconstriction |
| **#10**  **#11**  **#12**  **#13**  **#14**  **#15**  **#16** | #5 OR #6 OR #7 OR #8 OR #9  "Endovascular Procedures"[Mesh]  "endovascular procedure" OR "Intravascular Procedure"  "Intravascular Technique" OR "Endovascular Technique"  angiosurgery OR balloon OR stents OR dilatation OR transluminal OR tube OR clip  catheter OR fibrin glue OR guide wire OR implant OR needle OR revascularization  #11 OR #12 OR #13 OR #14 OR #15 |
| **#17** | #4 AND #10 AND #16 |

Supplemental Table 1: Search strategies for this study in PubMed.
