# Supplementary material for: Efficacy of endovascular therapy for cerebral vasospasm following aneurysmal subarachnoid hemorrhage: a systematic review and meta-analysis
Source: Front Neurol. 2024 Apr 23;15:1360511. doi: 10.3389/fneur.2024.1360511 (PMC11075757; doi:10.3389/fneur.2024.1360511)
Supplement: Supplementary file 2 [file Data_Sheet_2.docx]

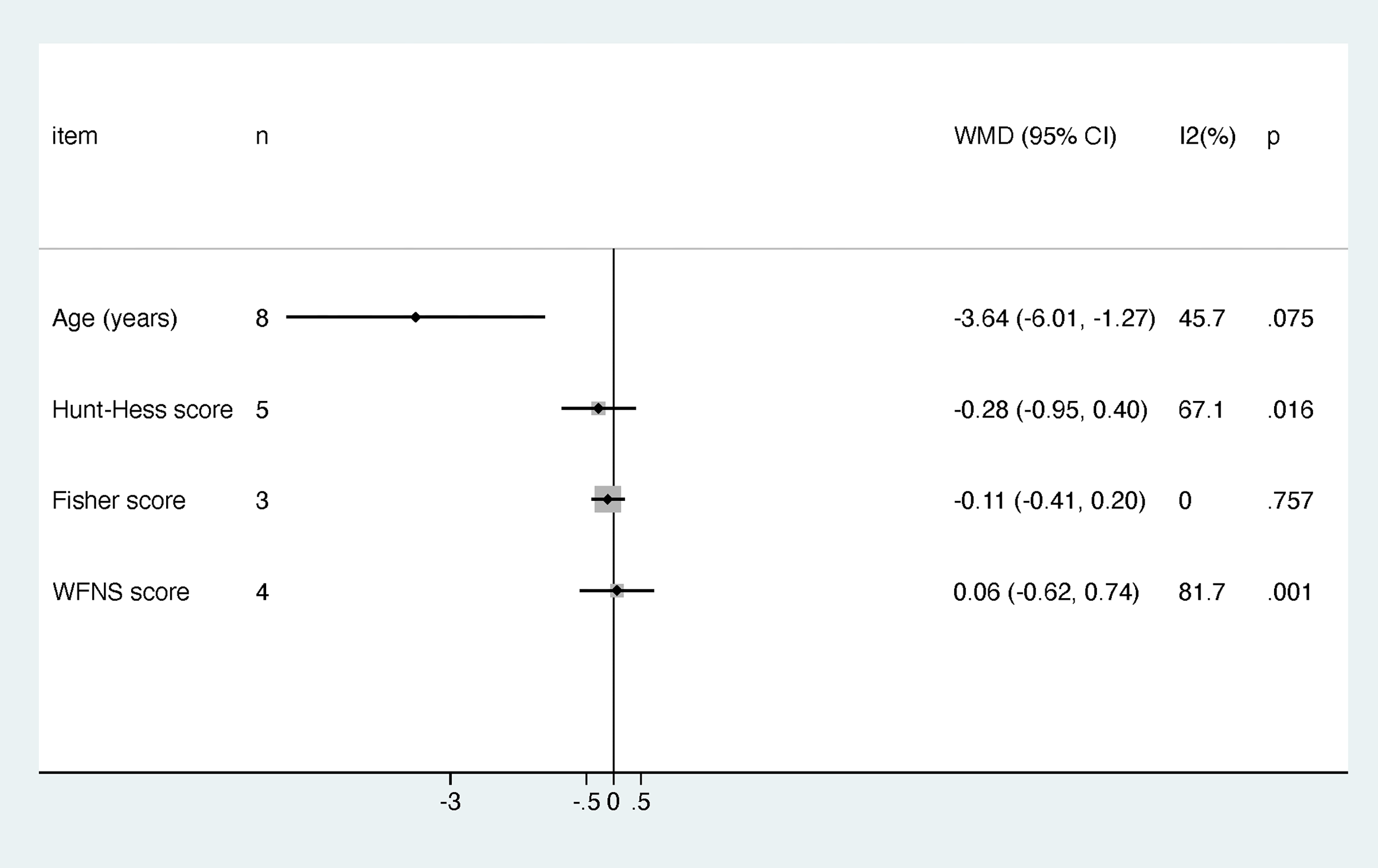

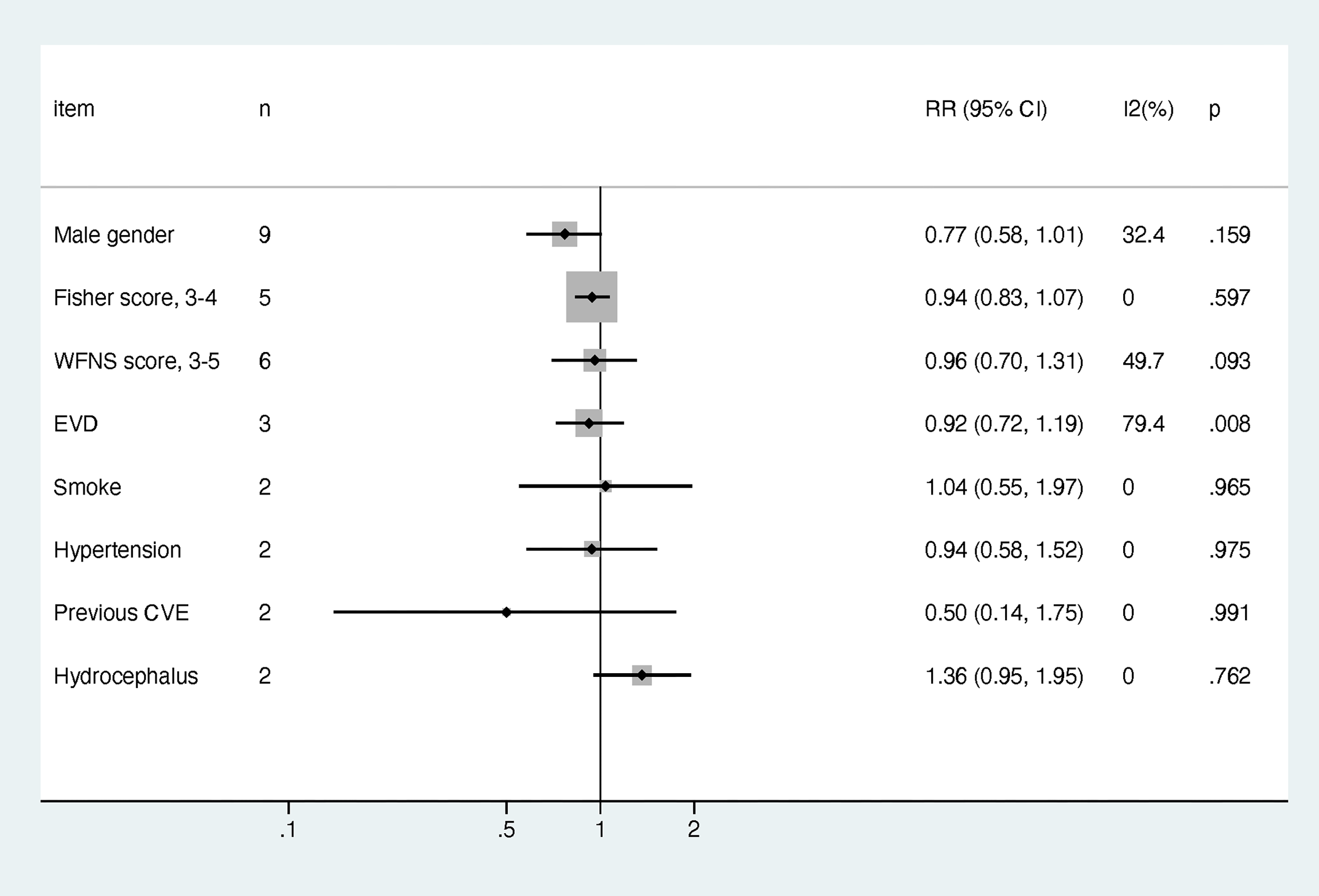


Supplemental Figure 1 Supplemental Figure 2


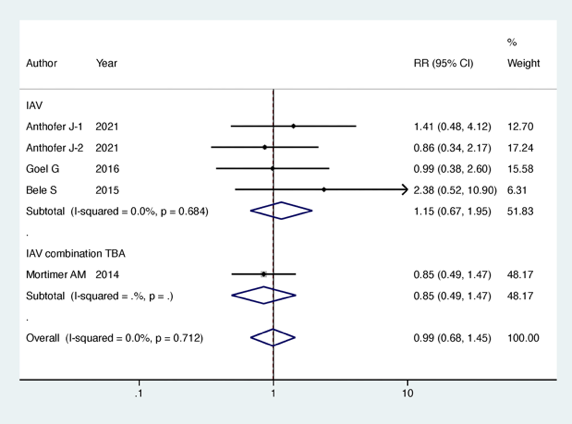

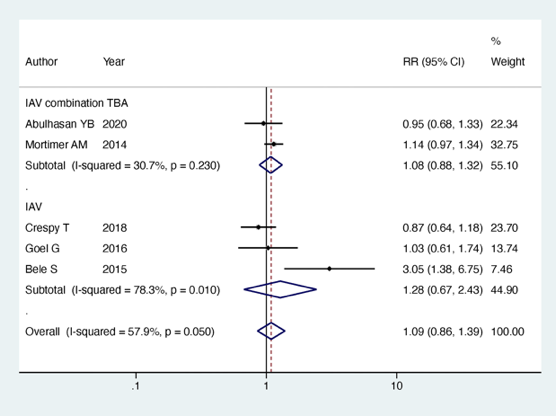


Supplemental Figure 3 Supplemental Figure 4


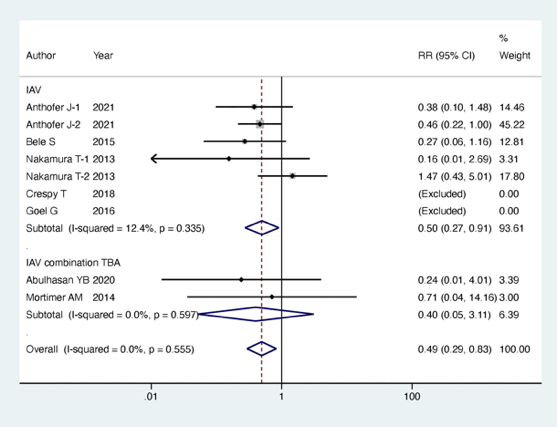

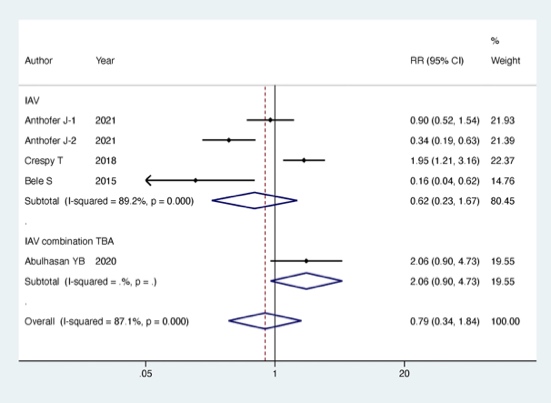


Supplemental Figure 5 Supplemental Figure 6


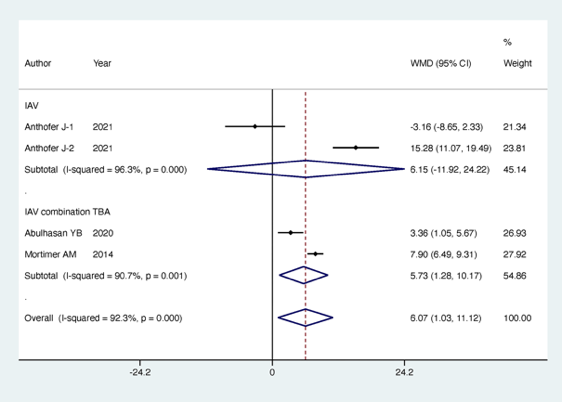


Supplemental Figure 7


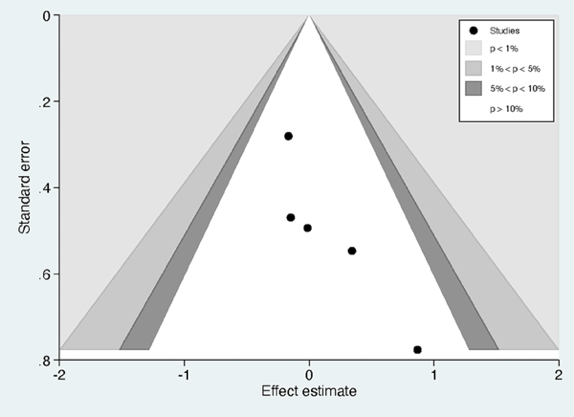

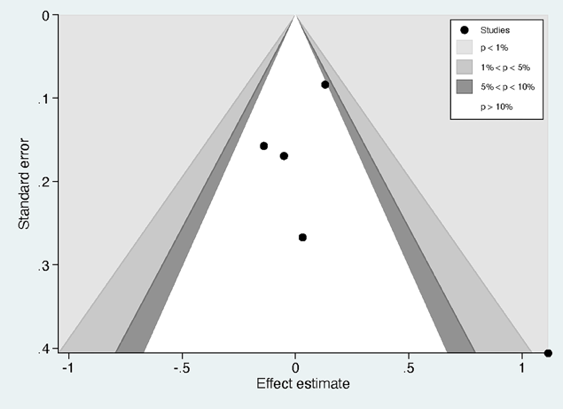


Supplemental Figure 8 Supplemental Figure 9


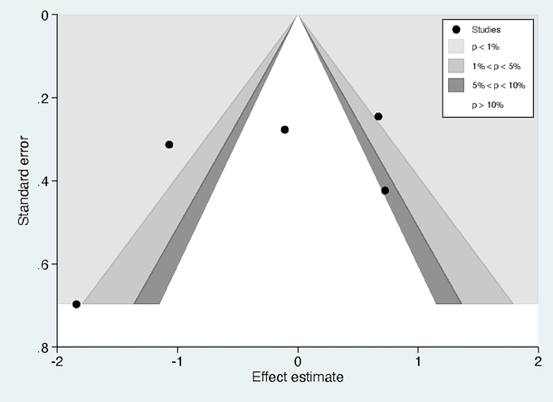

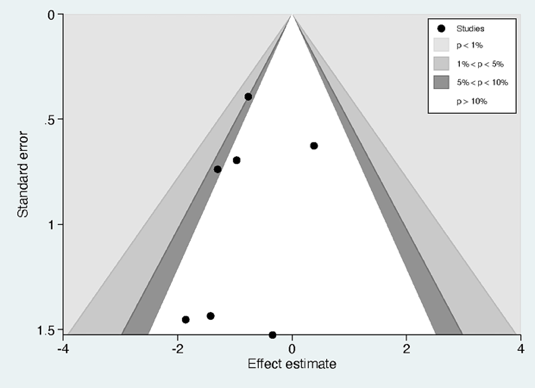


Supplemental Figure 10 Supplemental Figure 11
